# CTCF-Dependent Chromatin Bias Constitutes Transient Epigenetic Memory of the Mother at the *H19-Igf2* Imprinting Control Region in Prospermatogonia

**DOI:** 10.1371/journal.pgen.1001224

**Published:** 2010-11-24

**Authors:** Dong-Hoon Lee, Purnima Singh, Shirley Y. Tsai, Nathan Oates, Alexander Spalla, Claudio Spalla, Lucy Brown, Guillermo Rivas, Garrett Larson, Tibor A. Rauch, Gerd P. Pfeifer, Piroska E. Szabó

**Affiliations:** 1 Department of Molecular and Cellular Biology, Beckman Research Institute, City of Hope National Medical Centre, Duarte, California, United States of America; 2 Shared Recourses, Beckman Research Institute, City of Hope National Medical Centre, Duarte, California, United States of America; 3 Department of Molecular Medicine, Beckman Research Institute, City of Hope National Medical Centre, Duarte, California, United States of America; 4 Department of Biology, Beckman Research Institute, City of Hope National Medical Centre, Duarte, California, United States of America; Massachusetts General Hospital, Howard Hughes Medical Institute, United States of America

## Abstract

Genomic imprints—parental allele-specific DNA methylation marks at the differentially methylated regions (DMRs) of imprinted genes—are erased and reestablished in germ cells according to the individual's sex. Imprint establishment at paternally methylated germ line DMRs occurs in fetal male germ cells. In prospermatogonia, the two unmethylated alleles exhibit different rates of de novo methylation at the *H19/Igf2* imprinting control region (ICR) depending on parental origin. We investigated the nature of this epigenetic memory using bisulfite sequencing and allele-specific ChIP–SNuPE assays. We found that the chromatin composition in fetal germ cells was biased at the ICR between the two alleles with the maternally inherited allele exhibiting more H3K4me3 and less H3K9me3 than the paternally inherited allele. We determined genetically that the chromatin bias, and also the delayed methylation establishment in the maternal allele, depended on functional CTCF insulator binding sites in the ICR. Our data suggest that, in primordial germ cells, maternally inherited allele-specific CTCF binding sets up allele-specific chromatin differences at the ICR. The erasure of these allele-specific chromatin marks is not complete before the process of de novo methylation imprint establishment begins. CTCF–dependent allele-specific chromatin composition imposes a maternal allele-specific delay on de novo methylation imprint establishment at the *H19/Igf2* ICR in prospermatogonia.

## Author Summary

Allele-specific DNA methylation is considered the primary mark that distinguishes the parental alleles of imprinted genes. Whereas allele-specific chromatin also exists at imprinted genes in the soma, this has not been assessed in the germ line. It will be important to understand what extent the chromatin composition provides clues in the germ line to the erasure and establishment of methylation imprints. Our novel methods provide the sensitivity required to answer these questions. Our results argue that the erasure of the DNA methylation imprint is complete before, and therefore does not depend on, the erasure of allele-specific chromatin marks at the *H19/Igf2* imprint control region. Additionally, we show that incomplete erasure of the allele-specific chromatin is responsible for the delayed DNA methylation imprint establishment of the maternal ICR allele in prospermatogonia. The chromatin bias—the transient epigenetic memory of the mother—in fetal germ cells depends on functional CTCF insulator binding sites in this imprint control region.

## Introduction

Imprinted genes are epigenetically modified during germ cell development, such that their expression in somatic cells depends on the parent of origin [1], [2]. Allele-specific differential DNA methylation is associated with most imprinted genes [3]. Male or female-specific methylation of the germ line differentially methylated regions (DMRs) is inherited from the gametes, survives the global wave of demethylation during early embryogenesis and is faithfully maintained in somatic cells during the life of the individual. Deletion studies showed that some DMRs are critical for allele-specific monoallelic expression of imprinted genes [4]–[8]. The importance of DNA methylation in the establishment and maintenance of genomic imprinting has been demonstrated in mice in which DNA methyltransferase genes have been inactivated [9]–[12].

**Figure 1:**
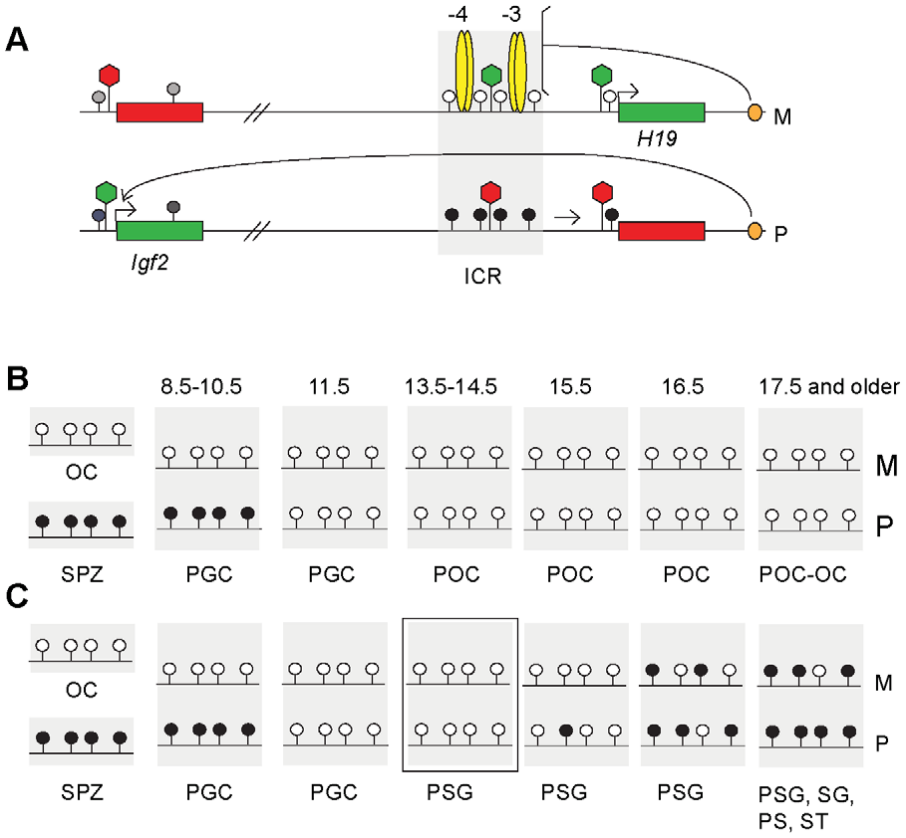
The imprint cycle at the *H19/Igf2* ICR. Schematic representation of epigenetic features at the *H19/Igf2* imprinted domain based on publications referenced in the Introduction. (A) The *H19/Igf2* imprinted domain in the soma. Maternal chromosome (M): unmethylated (white lollipops) ICR (shaded area) is inherited from the egg. CTCF protein (yellow ovals) at binding sites 1–2 and 3–4 at about −4 kb and −3 kb upstream of the *H19* transcription start site imparts insulator activity (bracket) between the *Igf2* promoters and the shared, downstream enhancers (orange oval). Paternal chromosome (P): methylated (black lollipops) ICR is inherited from the sperm, CTCF cannot bind, hence ICR has no insulator activity, *Igf2* promoters and enhancers can interact. Early in postimplantation development, the *H19* promoter is inactivated by an ICR-dependent mechanism (horizontal arrow). Active or repressive chromatin (green or red hexagon) is present at expressed or silent alleles of genes (green-red rectangles) and at respective alleles of the ICR. (B) Fate of the imprint in the female germ line. Methylation status of the ICR is depicted in the mature oocyte (OC), spermatozoon (SPZ), primordial germ cells (PGC) primary oocytes (POC) at gestational stages (in dpc). (C) Fate of the imprint in the male germ line. Methylation status is depicted in OC, SPZ and PGC as above and in prospermatogonia (PSG), spermatogonia (SG) pachytene spermatocytes (PS) and round spermatids (ST). The developmental stage under investigation is marked by a rectangle.

The paternally expressed insulin-like growth factor 2 (*Igf2*) and maternally expressed *H19* genes on mouse distal chromosome 7 [13] are coordinately expressed during embryonic development, due to shared tissue-specific enhancers (Figure 1A) [14], [15]. A paternally methylated germ line DMR between *Igf2* and *H19* [16]–[18] is responsible for monoallelic expression of both *H19* and *Igf2* [19]–[21], and therefore, is called an imprinting control region (ICR). The regulatory functions of the ICR depend on allele-specific DNA methylation. Inactivation of the *H19* promoter takes place in post-implantation development on the paternal chromosome and it depends on ICR methylation [22]. The ICR functions as a methylation regulated enhancer blocker [23]–[27]: CTCF protein [28]–[30] binds in the unmethylated maternal allele and insulates between the *Igf2* promoters and the shared enhancers. DNA methylation in the paternal allele inhibits CTCF binding, hence the ICR has no insulator activity, and the *Igf2* promoters and the enhancers can interact. Targeted mutagenesis of the CTCF binding sites in the mouse results in a loss of enhancer-blocking activity and increased DNA methylation in the mutant maternal chromosome [31]–[33]. CTCF binding in the ICR is the major organizer of chromatin composition in the maternal allele along the entire imprinted domain [34]–[36]. CTCF recruits active histone tail modification marks to the ICR and to the *H19* gene [34] and also recruits at a distance, Polycomb-mediated H3K27me3 repressive marks at the *Igf2* promoter and at the *Igf2* DMRs [34], [35].

CpG methylation at DMRs is reset during germ cell development: inherited gametic marks are erased in primordial germ cells (PGCs) followed by the establishment of new gametic marks in the female and male germ lines according to the individual's sex (Figure 1B and 1C). The umethylated versus methylated status of the *H19/Igf2* ICR in oocytes versus spermatozoa constitutes the female and male gametic mark. Methylation of the paternal allele is erased in female and male germ cells by 13.5 days post coitum (dpc) [37]–[40] (Figure 1B and 1C). In the female germ line the ICR remains unmethylated during fetal and postnatal stages of oogenesis (M and P alleles in Figure 1B). In male germ cells, the ICR methylation imprint is laid down between 15.5–17.5 dpc, and is almost fully established by 18.5 dpc [31], [41]. The germ line-specific processes that target differential methylation to the ICR are unknown but are entirely separate from the later somatic ICR functions of chromatin insulation and *H19* promoter silencing. CTCF binding is not required to establish an unmethylated ICR during oogenesis or a methylated ICR during spermatogenesis. The ICR that lacks functional CTCF binding sites is unmethylated in female fetal germ cells and ovulated oocytes but is methylated in perinatal spermatogonia [31], [32].

The timing of DNA methylation between the maternally and paternally inherited alleles (M and P alleles in Figure 1C) is different during spermatogenesis, methylation of the paternally inherited allele preceding that of the maternally inherited allele, implying that the two parental alleles can be distinguished from each other by the de novo DNA methylation machinery in the absence of DNA methylation [37], [39], [41], [42]. We sought to investigate the nature of this epigenetic memory in spermatogonia. We hypothesized that differences in CTCF protein binding and/or chromatin composition between the paternally or maternally inherited alleles are responsible for discriminating between the parental alleles in the male germ line. We based this hypothesis on previous observations: We have shown that migratory PGCs exhibit strict imprinted maternal allele-specific *H19* expression at 8.5 dpc, and paternal allele-specific *Igf2* expression at 10.5 dpc [43]. Expression of *H19* and *Igf2* becomes biallelic by the early post-migratory stage of 11.5 dpc [43], [44] and remains biallelic during fetal and postnatal stages of spermatogenesis [44]. Because parental allele-specific expression of both *H19* and *Igf2* depends on CTCF insulator binding in the maternally inherited ICR allele [31]–[33], CTCF binding in the ICR must be maternal allele-specific in migratory PGCs and biallelic or missing at later stages of spermatogenesis. It is not known if allele-specific chromatin differences exist in PGCs or if these become erased at the time when DNA methylation marks are erased at DMRs. CTCF binding, however, likely organizes the chromatin composition of the ICR in the maternal allele in PGCs, similarly to its role in somatic cells [34]. The allele-specific chromatin difference may also need to be erased in postmigratory spermatogonia, for example H3K4 methylation would be removed from the maternal allele, because H3K4 methylation is not permissive for de novo DNA methylation [45]. Erasure of chromatin marks may occur synchronously with the global dynamic changes of chromatin reorganization that take place in germ cells around mid-gestation [46]–[48]. If allele-specific chromatin marks are not fully erased in prospermatogonia after methylation imprint erasure is complete, they may influence the rate of de novo methylation. We can test this possibility directly and specifically by perturbing the chromatin bias of the ICR in prospermatogonia. After maternal transmission of the ICR CTCF site mutations [31], [34] we expect to find loss of allele-specific differences in chromatin composition and methylation establishment in prospermatogonia.

Using allele-specific chromatin immunoprecipitation single nucleotide primer extension (ChIP-SNuPE) assays we found that in normal prospermatogonia the chromatin composition was biased between the two alleles after complete erasure of CpG methylation. The CTCF site mutant maternal ICR allele, however, no longer exhibited those allele-specific chromatin differences and delayed methylation establishment. Our data suggest that CTCF dependent allele-specific chromatin composition gives de novo methylation imprint establishment an allele-specific bias at the *H19*/*Igf2* ICR.

## Results

To assess DNA methylation and chromatin in prospermatogonia and primary oocytes, we obtained high purity germ cell populations using the TgOG2 transgenic mouse line [43], in which the enhanced green fluorescent protein (EGFP) is expressed from the *Pou5f1* promoter in gestational-stage germ cells. We have shown earlier using flow cytometry that in TgOG2 transgenic embryos the EGFP positive cell population is highly synonymous at the premigratory and early postmigratory stages with the populations staining positive for other PGC markers, alkaline phosphatase 2 and stage specific embryonic antigen (SSEA) [43]. Immunocytochemistry of the fetal germ cell populations before and after cell sorting using anti-DDX4 antibody confirmed that flow sorting resulted in a high level of enrichment (Figure S1). Additionally, bisulfite sequencing of the KvDMR1 [6], [49] correctly only detected unmethylated chromosomes in fetal germ cells at 13.5, 15.5 and 17.5 days post coitum (dpc) (Figure S2). This maternally methylated DMR is unmethylated in fetal germ cells at 12.5–13.5 dpc [46] and only becomes methylated postnatally, in growing oocytes [50]. Fully methylated chromosomes (methylated maternal allele) would indicate contamination from the somatic cells.

### Bisulfite sequencing confirms a delay of imprint establishment at the ICR in the maternally inherited allele in prospermatogonia

**Figure 2:**
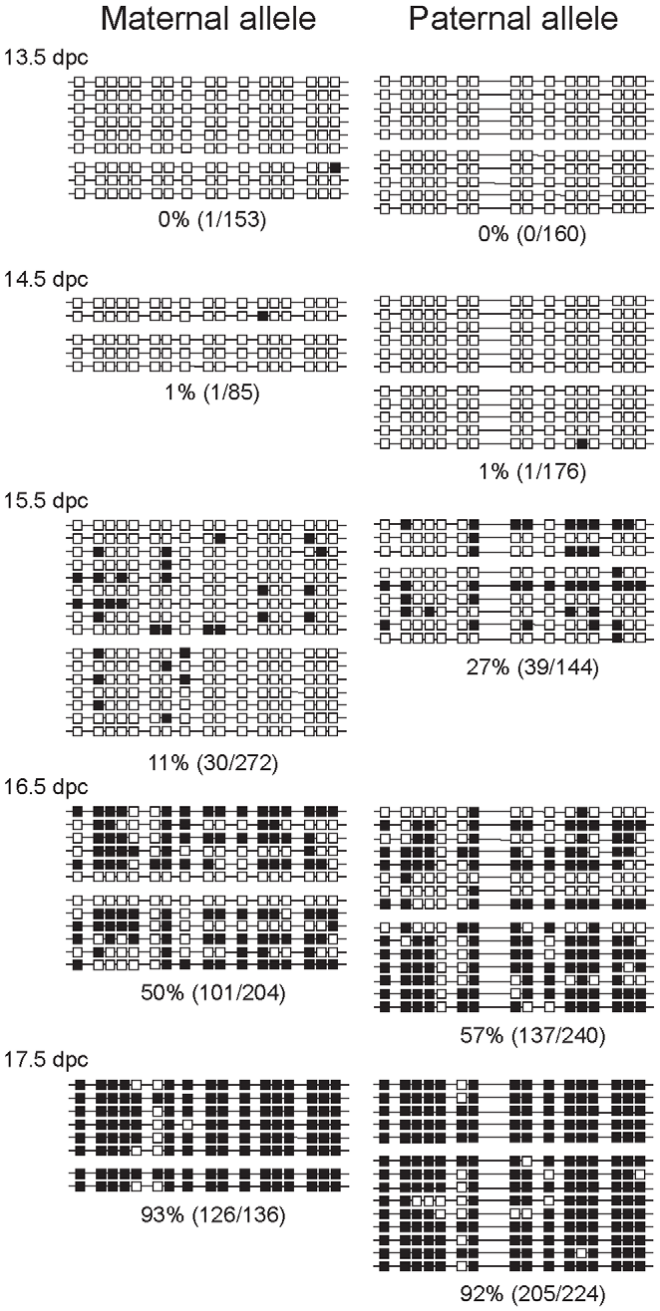
Methylation dynamics at the ICR in normal prospermatogonia. Bisulfite sequencing results are shown at fetal stages (in dpc). Prospermatogonia of CS X OG2 fetuses were analyzed. Unmethylated CpGs (white squares) and methylated CpGs (black squares) are shown along independent chromosomes (horizontal lines). Groups of chromosomes were derived from the same bisulfite reaction. CTCF sires 1 and 2 of the ICR are included in the analyzed region. CpG site 8 is polymorphic and is only present in the CS type allele. The percentage of methylated CpGs (methylated CpG/total CpG) at each developmental stage is indicated for each allele.

In prospermatogonia, the paternally inherited ICR allele becomes methylated earlier than the maternally inherited allele in reciprocal crosses between C57BL/6J (B6) and JF1 [39], [41]. Similarly, when the ICR carries the B6 type allele in the maternal allele and the CAST/Ei type allele in the paternal allele, the B6 type maternal allele is delayed compared to the CAST/Ei type paternal allele in prospermatogonia between 14.5 and 18.5 dpc [37]. We tested the reciprocal situation when the CAST/Ei type ICR allele is inherited from the mother and the B6 type allele is inherited from the father. Females of FVB/NJ.CAST/Ei(N7), a distal chromosome 7 partial congenic strain for CAST/Ei (CS) [31] were mated with TgOG2 homozygous transgenic males [43] resulting in CS X OG2 fetuses. We isolated male and female germ cells from 13.5, 14.5, 15.5, 16.5 and 17.5 dpc gonads. We performed two or more independent bisulfite conversion reactions for each sample and sequenced at least twelve clones of each sample. A single nucleotide polymorphism in the CS strain was used to identify the parental alleles. We confirmed previous observations [37]–[40] that DNA methylation erasure is complete by 13.5–14.5 dpc at the ICR (Figure 2 and Figure S3). We found that primary oocytes exhibited no methylation of the ICR region between 13.5 and 16.5 dpc (Figure S3A) and prospermatogonia attained CpG methylation gradually (Figure 2) between 15.5 dpc and 17.5 dpc as expected [37], [39]. We confirmed that the maternal allele (CS type) was delayed compared to the paternal allele (B6 type) in CS X OG2 prospermatogonia (Figure 2) similar to the reciprocal B6 X CS situation [37]. Regardless of mouse strains used, there exists a time gap in methylation imprint establishment between the two chromosomes depending on the inheritance from the mother or father (M and P alleles in Figure 1) during spermatogenesis [37], [39], [41], [42]. Therefore, the two parental alleles must be distinguished from each other in 13.5–14.5 dpc prospermatogonia by epigenetic means other than DNA methylation.

### CTCF site mutations abolish delayed methylation imprint establishment of the maternal ICR allele

**Figure 3:**
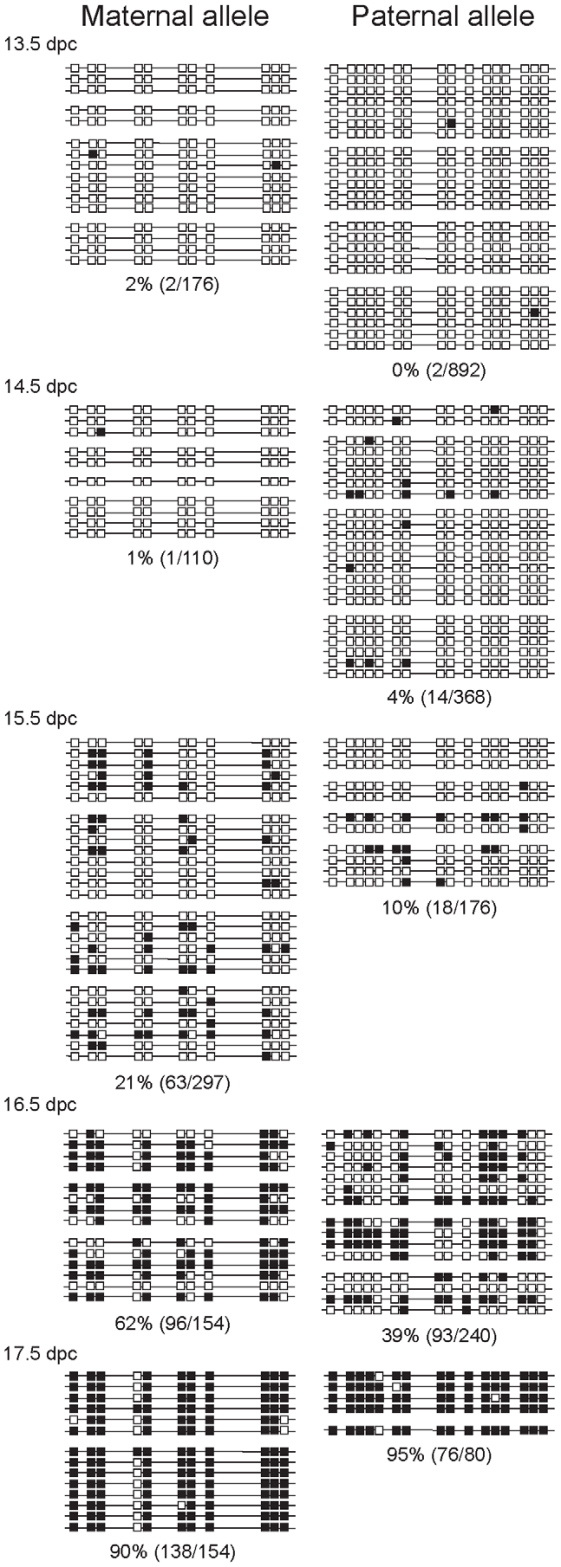
Methylation dynamics at the ICR in ICR CTCF site mutant prospermatogonia. Bisulfite sequencing results of prospermatogonia from CTCFm X OG2 fetuses are shown. CpG sites 4–5 and 12–13 had been eliminated in the maternal allele by the CTCF site mutations. Other details are as in Figure 2.

We tested the hypothesis that functional CTCF binding sites in the maternally inherited *H19*/*Igf2* ICR allele are responsible for the delayed methylation of the maternally inherited, compared to the paternally inherited allele in male germ cells. Female mice homozygous for CTCF site mutations (−/−) [31] were mated with TgOG2 homozygous transgenic males [43] (wild type ICR). In the resulting CTCFm X OG2 fetuses, the maternally inherited ICR allele was mutant, lacking functional binding sites. Germ cells were collected at 13.5, 14.5, 15.5 and 16.5 dpc. Bisulfite DNA sequencing was performed on agarose-embedded germ cells as described before [31] according to Olek et al. [51]. Nucleotide changes, introduced with the mutations aided discrimination between the mutant and wild type alleles. We found that due to the ICR CTCF site mutations the maternally inherited mutant allele did not lag behind the paternal allele in male germ cells (Figure 3). The increased rate of methylation in the CTCF site mutant ICR maternal allele (Figure 3) compared to the normal ICR allele (Figure 2) was statistically significant. At 15.5 days the p-value = 0.0014 and at 16.5 days the p-value  = 0.0183 according to Fisher's exact test. This argues that intact CTCF protein binding sites in the ICR are required for the transient epigenetic memory that delays methylation of the maternally inherited allele during male fetal germ cell development. When the paternal ICR carried the CTCF site mutations in the control OG2 X CTCFm male germ cells (Figure S4), its rate of methylation was similar to the normal paternal allele in the CS X OG2 cross (Figure 2), indicating that simply having less CpG sites in the CTCF site mutant ICR is not sufficient to alter the rate of methylation. The control female germ cells did not attain methylation in the mutant allele (Figure S3B).

The mutant maternal allele was, unexpectedly, more prone to methylation than the wild-type paternal allele in the same cell. The wild type paternal and mutant maternal alleles are different in two respects, in the strain and in the presence or absence of the CTCF site mutations. The best comparison can be made when the CTCFm allele is compared between paternal and maternal inheritance. The methylation levels of these chromosomes, indeed, were very similar at 14.5 and 15.5 dpc (Figure 3 and Figure S4). We noted that the sum level of methylation in the two alleles did not change between the wild type and CTCF site mutant prospermatogonia, indicating perhaps that the two alleles are in competition for a methylation inducing factor that has limited concentration at 15.5–16.5 dpc.

### Chromatin composition at the ICR is biased between parental alleles in 13.5 and 14.5 dpc prospermatogonia

We considered the possibility that differences in CTCF binding and chromatin composition between the paternally or maternally inherited alleles might be responsible for discriminating between the parental ICR alleles in the male germ line. If this is correct, we would expect in spermatogonia a slight bias in chromatin composition between the maternally and paternally inherited alleles at the ICR such that the paternally inherited allele would be more permissive to DNA methylation. We developed ChIP-SNuPE assays based on mass spectrometry Sequenom allelotyping [36], [52] to distinguish allele-specific incorporation of ddNTPs into the SNuPE primer based on differences in molecular mass at sites of single nucleotide polymorphisms (SNPs) between 129 or OG2 and CS mouse genomic sequences along the *H19/Igf2* ICR [34]. The Sequenom assay used SNPs at two halves of the ICR at −4 kb and −3 kb distances from the *H19* transcriptional start site. Both assays were rigorously quantitative, as shown by DNA mixing experiments (Figure S5A). The number of fetal germ cells is limiting for ChIP assays, we can obtain 100,000–300,000 germ cells per dissection. We decided to use 100,000 germ cells per ChIP. We validated the ChIP-SNuPE assays using 100,000 129 X CS mouse embryo fibroblasts (MEFs). We found that CTCF binding and active chromatin (H3K4me2 enrichment) was highly specific to the maternal allele in the ICR whereas repressive chromatin (H3K9me3) was highly specific to the paternal allele in MEFs (Figure S6A) as we previously reported using large number of the same 129 X CS MEF cells [34]. We found that the assay correctly measured 50% 129 and CS alleles in the input chromatin samples for MEFs (Figure S6A) and for CS X OG2 and CTCFm X OG2 fetal germ cells (Figure S6C).

**Figure 4:**
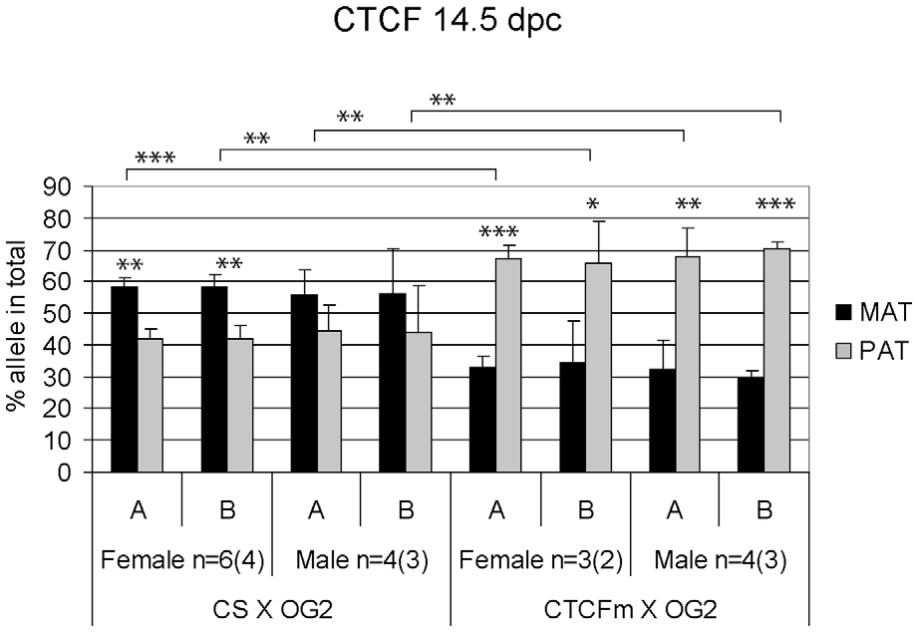
Allele-specific bias in CTCF binding chromatin at the *H19/Igf2* ICR in 14.5 dpc fetal germ cells. Female and male germ cell chromatin was precipitated from 14.5 dpc CS X OG2 and CTCFm X OG2 fetuses with the anti-CTCF antibody. Allele-specific enrichment in the immunoprecipitated chromatin was assessed at the *H19/Igf2* ICR −4 kb and −3 kb regions (A and B, respectively) using ChIP-SNuPE assays. The number of ChIP reactions (n) is indicated and the number of independent germ cell pools/chromatin preparations is given in parentheses. Average maternal (MAT) and paternal (PAT) allele contributions are shown with standard deviations. Statistical significance of the difference between alleles and between wild type and mutant samples was evaluated using Student T-test (p values are shown by asterisks: <0.001***; <0.01**; <0.05*).

**Figure 5:**
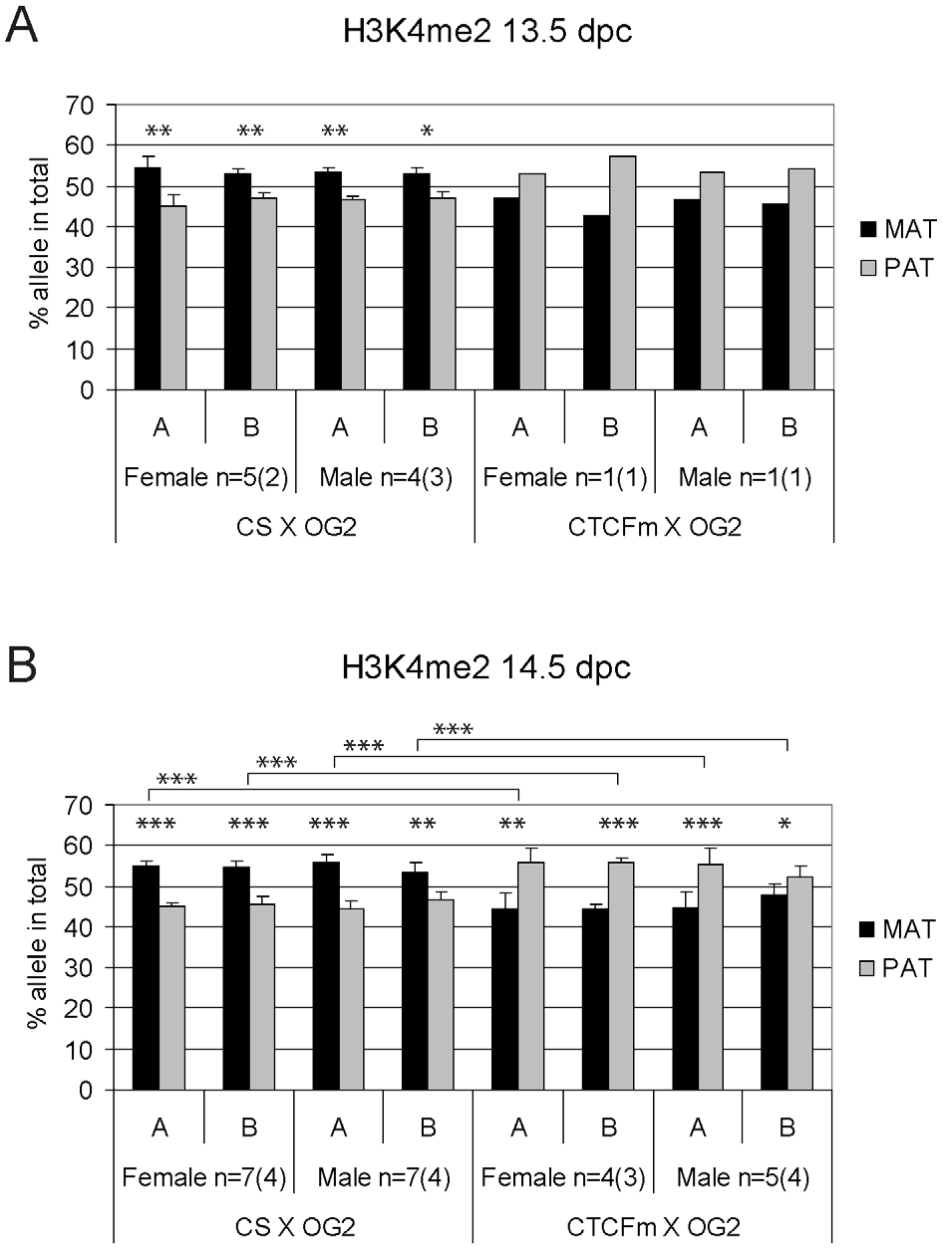
Allele-specific bias in H3K4me2 enrichment at the *H19/Igf2* ICR in fetal germ cells. ChIP-SNuPE Sequenom assay results of H3K4me2-precipitated (A) 13.5 dpc and (B) 14.5 dpc fetal germ cell chromatin are shown. Other details are as in Figure 4.

**Figure 6:**
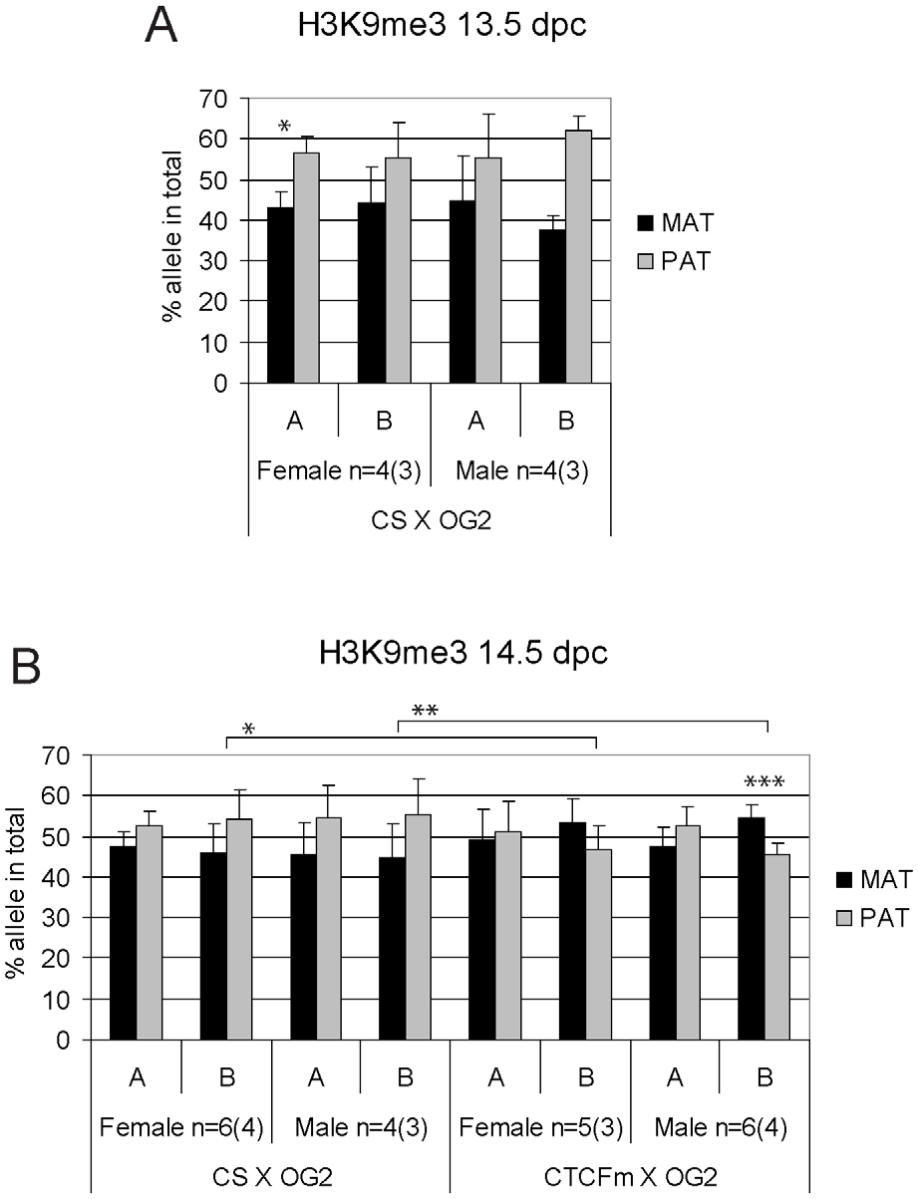
Allele-specific bias in H3K9me3 enrichment at the *H19/Igf2* ICR in fetal germ cells. ChIP-SNuPE Sequenom assays results of H3K9me3-precipitated (A) 13.5 dpc and (B) 14.5 dpc fetal germ cell chromatin is shown. Other details are as in Figure 4.

We isolated male and control female germ cells from 13.5 and 14.5 gonads from the CS X OG2 mouse cross and performed ChIP-SNuPE assays using 100,000 germ cells per ChIP reaction. The control, nonspecific IgG-precipitated chromatin samples did not exhibit a clear pattern of allele-specific skewing (Figure S6B). The results did not show consistency between the −4 kb and −3 kb regions (A and B regions, respectively) or between the 13.5 and 14.5 dpc stages. Specific antibodies, on the other hand gave reproducible results using germ cell chromatin (Figure 4, Figure 5, Figure 6). CTCF binding was slightly biased toward the maternal ICR allele in male and female germ cells at 14.5 dpc (Figure 4). CTCF binding in the paternal allele would likely be inhibited by DNA methylation in PGCs similarly to somatic cells [23], [24], [27], but not in fetal prospermatogonia at 13.5–14.5 dpc in the lack of DNA methylation. The slight maternal bias is consistent with the possibility that allele-specific CTCF binding is not completely erased at 14.5 dpc, after DNA methylation erasure had been completed. The total level of CTCF binding at the ICR was very low in germ cells at 14.5 dpc compared to MEFs (Figure S7). This suggests that CTCF has been almost completely removed from both ICR alleles in germ cells by 14.5 dpc, consistent with biallelic *Igf2* expression in the absence of insulation [31]–[33], [43]. The almost complete lack of CTCF binding, however is not due to the absence of CTCF from prospermatogonia at these stages. This would be expected based on that CTCF and CTCFL (BORIS) proteins exhibit mutually exclusive expression in adult male germ cells, round spermatids and spermatocytes, respectively [53] and that CTCFL is expressed in 14.5 dpc prospermatogonia [54]. It is not known whether CTCF is expressed in embryonic and fetal germ cells. We addressed this question by performing immunocytochemistry with anti-CTCF antibody using fetal germ cells (Figure S8). We found that CTCF staining in male and female germ cells was similar to that of control gonadal somatic cells at 12.5 dpc and 14.5 dpc. The mutually exclusive expression of CTCF and CTCFL, therefore, does not apply in germ cells at 14.5 dpc. CTCF may be inhibited to bind in the ICR at these stages because of changes in its covalent modifications [55], cofactors, or due to an RNA-dependent mechanism [56].

**Figure 7:**
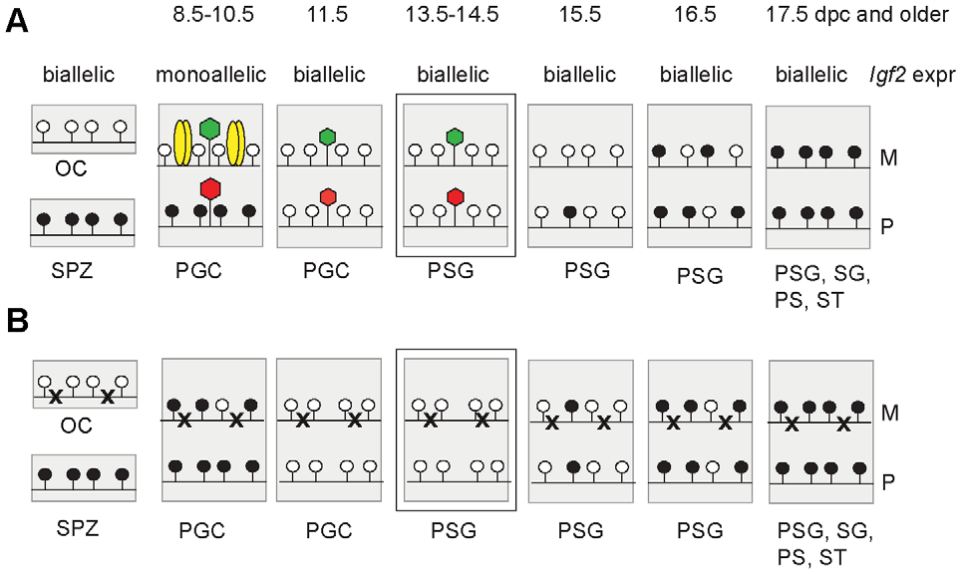
Model. Functional CTCF sites are required for chromatin bias and delayed methylation of the maternally inherited ICR allele. Expected CTCF binding and chromatin composition is depicted in primordial germ cells (PGC). Observed chromatin bias is depicted in prospermatogonia (PSG). Other details are as Figure 1. The developmental stages are indicated above in dpc. (A) Imprint establishment of the ICR in the normal male germ line. Chromatin bias is observed in the normal ICR between the parental alleles in the absence of CpG methylation at 13.5–14.5 dpc. (B) Imprint establishment at the CTCF site mutant ICR in the male germ line. CTCF cannot bind in the maternal allele in PGCs because of the mutations (x) or in the paternal allele because of CpG methylation. The chromatin bias, found in normal cells, is no longer observed between parental alleles in the mutant cells at 13.5–14.5 dpc and the maternal allele's methylation is not delayed at 15.5–17.5 dpc.

We found a slight (∼10%), but reproducible bias in the H3K4me2 levels toward the maternally inherited allele in male and female germ cell ChIP samples at 13.5 and 14.5 dpc (Figure 5). The bias was present in the ICR at −3 kb and −4 kb positions. H3K4me2 enrichment in germ cells was similar to the level found in MEFs (Figure S9), suggesting that the ICR had not been stripped of this mark at 13.5–14.5 dpc. H3K9me3 was reciprocally biased: the paternally inherited allele exhibited about 10% higher enrichment at 13.5 and 14.5 dpc (Figure 6). The allele-specificity of the bias for H3K4me2 and H3K9me3 in 13.5 dpc germ cells was in agreement with the somatic pattern (Figure S6A), being maternal and paternal specific, respectively, suggesting that it originates in premigratory PGCs (Figure 7A). The amplitude of the bias, however, was smaller than in the soma, consistent with the possibility that the chromatin differences are being erased in germ cells around mid-gestation and only the remnants of the allele-specific differences can be detected at 13.5–14.5 dpc. H3K9me3 levels at the ICR, however, were very low in germ cells at these stages (not shown), consistent with the possibility that similarly to CTCF but unlike H3K4me2 this mark is almost completely removed by 13.5 dpc.

The allele-specific bias of H3K4me2 in 14.5 dpc germ cells was present with only trace amounts of CTCF binding (Figure S7) in the ICR. We concluded that the H3K4me2 histone mark could be a potential candidate that provides the epigenetic memory of the mother at the ICR in 13.5–14.5 dpc prospermatogonia in the absence of CpG methylation.

### CTCF site mutations abolish parental allele–specific chromatin bias at the ICR in prospermatogonia

CTCF binding is essential for the maternal allele's chromatin composition along the *H19*/*Igf2* imprinted domain in the soma [34]. We decided to analyze if CTCF binding site mutations abolish the enrichment bias of histone covalent modifications between the maternally and paternally inherited alleles in fetal male germ cells. Female mice homozygous for the CTCF site mutations [31] were mated with TgOG2/TgOG2 transgenic males [43]. In the resulting CTCFm X OG2 fetuses, the maternally inherited allele was mutant, lacking functional binding sites. Male and control female germ cells were collected at 13.5 and 14.5 dpc and ChIP was performed with 100,000 germ cells per reaction using the H3K4me2 and H3K9me3 antibodies. Allele-specific precipitation was assessed using ChIP-SNuPE Sequenom assays that can distinguish the CTCF site mutation sites from the normal allele at CTCF binding sites 1 and 3 (at −4 kb and −3 kb positions, respectively) in the ICR. Each assay was rigorously quantitative, as shown by DNA mixing experiments (Figure S5B).

Contrary to what we found in CS X OG2 fetal germ cells, CTCF did not exhibit a slight bias toward the maternally inherited allele but instead a strong bias toward the paternal allele in 14.5 dpc CTCFm X OG2 germ cells (Figure 4). The reduction of maternal-allele specificity is consistent with impaired binding of CTCF to the mutant sites in the maternal allele. The paternal allele-specificity is likely due to the potential of CTCF binding in the paternal allele in the lack of methylation at 13.5–14.5 dpc. Using gel shift competition assays [31] and in vivo ChIP analysis [34] we have shown previously that the CTCF site mutations completely abolished CTCF binding in the ICR sequences. The fact that we do not measure a complete lack of maternal allele-specific CTCF binding in 14.5 dpc CTCFm X OG2 germ cells is most likely due to the limitation of the assay at extremely low copy numbers (Figure S7).

H3K9me3 was slightly paternally biased at 13.5–14.5 dpc in CS X OG2 germ cells (Figure 6) but was not consistently biased in CTCFm X OG2 germ cells at 14.5 dpc (Figure 6B). We observed a switch from a slight maternal- to a slight paternal H3K4me2 bias at −4 kb and also at −3 kb along the ICR (Figure 5B) suggesting that intact CTCF binding sites are required for distinguishing the maternal allele by H3K4 dimethylation in male and female germ cells at 14.5 dpc.

## Discussion

This is the first study addressing the chromatin composition of a DMR at any imprinted region in gestational stage germ cells. We tested the hypothesis whether the epigenetic memory of the father and the mother exists in fetal germ cells in the form of an allele-specific bias of chromatin composition after the erasure of the DNA methylation imprint at the *H19*/*Igf2* ICR. We found that the chromatin composition was biased at the ICR between the two alleles in fetal germ cells and this bias depended on functional CTCF insulator binding sites in the ICR. The CTCF site mutant maternal ICR allele no longer exhibited delayed methylation establishment. Our data suggest that CTCF dependent allele-specific chromatin composition gives de novo methylation imprint establishment a maternal allele-specific delay at the *H19*/*Igf2* ICR in prospermatogonia. A more general implication of our results is that the erasure of the allele-specific chromatin imprints is not fully synchronized with the erasure of CpG methylation at DMRs.

### Erasure of allele-specific chromatin marks follows the erasure of DNA methylation

We hypothesized that chromatin differences exist between parental alleles of DMRs in PGCs at the time of monoallelic expression of imprinted genes and that these chromatin differences are erased in the germ line. It would be extremely challenging technically to assess allele-specific chromatin in migratory PGCs because of the very low germ cell numbers at those stages. We found, however, evidence that parental allele-specific chromatin bias exists in the H3K4me2 and H3K9me3 residues in postmigratory germ cells at the *H19/Igf2* ICR at 13.5 and 14.5 dpc; thus the erasure of allele-specific chromatin lags behind the erasure of DNA methylation at the *H19*/*Igf2* ICR (Figure 7). The erasure of allele-specific chromatin at the ICR, therefore, is not required for the erasure of DNA methylation imprint.

It will be interesting to investigate the mechanism of how allele-specific chromatin marks are erased at DMRs. It is important to note that fetal germ cells do not divide after 13.5 dpc: spermatogonia enter mitotic arrest whereas primary oocytes arrest at the diplotene phase of meiosis, therefore, a passive loss of chromatin marks at DMRs is possible only before 13.5 dpc. DMR chromatin erasure might be linked with global chromatin remodeling events [46]–[48] around mid-gestation. The mechanism of global chromatin remodeling in PGCs is not known but is speculated to be mediated by chromatin chaperons [46]. We found that the rate of erasure at the ICR was different for the H3K4me2 and H3K9me3 marks. H3K4me2 overall enrichment appeared to hold on longer whereas H3K9me3 was largely removed by 14.5 dpc. This difference suggests that chromatin mark erasure at DMRs likely occurs by specific chromatin modifying enzymes, such as histone demethylases and does not involve nucleosome removal. Overall H3K4me2 erasure is likely completed at a later stage during spermatogenesis, because H3K4 dimethylation is absent at the ICR in postnatal male germ cells spermatocytes, round spermatids and elongating spermatids [57].

### CTCF sites are responsible for chromatin differences between alleles and for delayed methylation of the maternal ICR allele in prospermatogonia

We confirmed previous observations [37]–[40] that DNA methylation erasure at the ICR is complete by 13.5–14.5 dpc. If epigenetic memory existed of the mother or father in prospermatogonia that could distinguish the parental alleles at this time, it had to be distinct from CpG methylation. 5-hydroxy-methyl C (5hmC) emerges as a second covalent DNA modification with potential for epigenetic regulation [58], [59]. Because bisulfite sequencing recognizes not only 5mC but also 5hmC [60], our data are consistent with the absence of epigenetic memory of a parent in the form of both of these DNA covalent modifications at 13.5–14.5 dpc. Prospermatogonia attained CpG methylation at the ICR gradually between 15.5 dpc and 17.5 dpc with an allele-specific bias in the rate of methylation, confirming that there was epigenetic distinction between the parental alleles. Methylation of the maternal allele was slower than the paternal allele in normal spermatogonia, but not in CTCFm X OG2 spermatogonia where the ICR CTCF sites were mutant, arguing that functional CTCF sites are required in the maternal allele for its delayed methylation. We found maternally biased CTCF binding in the ICR at 13.5–14.5 dpc, consistent with the possibility that a bias in CTCF binding may provide the epigenetic memory of the mother. However, CTCF binding was only at trace levels suggesting that CTCF is not likely the factor that physically delays DNA methylation in the maternal allele at 15.5 dpc. Our data are in agreement with the model (Figure 7) that CTCF binding in the maternal allele organizes allele-specific chromatin differences at the ICR in PGCs and these chromatin marks are erased with a slower rate than the rate of DNA methylation erasure. The remnants of chromatin differences at 13.5–14.5 dpc may simply reflect their history and may not be responsible for the methylation bias. Alternatively, these marks may constitute the epigenetic memory that distinguishes the parental alleles for de novo methylation, commencing at 15.5 dpc. Indeed, in the absence of CTCF binding in the mutant ICR there was no maternal-allele-specific H3K4me2 bias and the methylation rate of the maternal allele was not delayed compared to the paternal allele in prospermatogonia, giving support to our model (Figure 7).

### Chromatin difference constitutes the transient epigenetic memory of the parental alleles in prospermatogonia

With the erasure of genomic imprints around mid-gestation the female and male germ lines are preparing for the establishment of the new imprints according to the individual's sex. It will be important to find out how the chromatin composition provides clues to the methylation imprint establishment. The chromatin composition at the paternally methylated DMRs is expected to be permissive to de novo methylation in 15.5–18.5 dpc spermatogonia and refractory to de novo methylation in growing oocytes. Our results argue that the erasure of chromatin clues at the H3K4me2 and H3K9me3 residues overlaps with the initiation phase of de novo methylation imprint establishment at the ICR and the incomplete erasure of these allele-specific chromatin marks can affect the rate of the new methylation imprint establishment in prospermatogonia.

Histone covalent modifications could take active part in or influence DNA methylation imprint establishment in the germ line, based on studies describing the interplay between histone methylation and DNA methylation. Histone H3K9 methylation controls DNA methylation in *Neurospora crassa* [61], [62] and in *Arabidopsis thaliana* [63], [64]. Histone lysine methylation by Suv39h1 is required for DNA methylation at the pericentric heterochromatin in mice [65]. Our genetic system [31], [34] is uniquely suited for asking the question whether disturbing the bias in chromatin composition specifically at the *H19/Igf2* locus would abolish the bias of methylation imprint establishment at the ICR in male fetal germ cells. H3K9me3 was biased toward the paternal ICR allele at 13.5 dpc, and H3K4me2 was biased towardF the maternal allele at 13.5–14.5 dpc in prospermatogonia. In the absence of paternal H3K9me3 bias in the 13.5 dpc CTCFm X OG2 prospermatogonia, the paternal allele's methylation rate was reduced, whereas in the lack of maternal H3K4m2 bias in 13.5–14.5 dpc prospermatogonia, the maternal allele's methylation rate increased. These findings suggest that chromatin composition differences between the parental alleles may influence the rate of their de novo methylation at the ICR.

Male and female germ cells behaved similarly with respect to the dynamics of the overall levels and the allele-specificity of H3K4m2 and H3K9me3 enrichment at the *H19/Igf2* ICR at 13.5 and 14.5 dpc, yet methylation imprint establishment was affected only in male germ cells. The maintenance of the unmethylated state of the ICR in fetal female germ cells was not affected by the chromatin bias. It is likely that the chromatin composition provides clues to exclude or target the de novo DNA methyltransferase complex to DMRs. Because Dnmt3a and Dnmt3L are specifically expressed in male versus female fetal germ cells [66]–[69], these would be affected by allele-specifically biased chromatin in prospermatogonia but not in primary oocytes.

The level of H3K4me2 bias toward the maternal allele was about 10% and thus was similar to the average 15% maternal allele-specific bias in delay of DNA methylation at 15.5 dpc. The H3K4me2 bias between the parental alleles existed in the lack of DNA methylation and with only a trace amount of CTCF binding in the ICR. We concluded that the H3K4me2 histone mark could provide the epigenetic memory of the mother in prospermatogonia at 13.5–14.5 dpc that delays de novo CpG methylation in the maternal ICR allele. Significantly, H3K4 demethylase KDM1B is required at certain DMRs for the establishment of maternal methylation imprints in oocytes [45], indicating that methylated H3K4 is refractory to DNA de novo methylation. Additionally, the DNA de novo methylation cofactor, Dnmt3L [70] requires a DNA substrate in association with histones containing unmethylated H3K4 [71].

Two other paternally methylated DMRs, the *Rasgrf1* DMR and the *Dlk1/Gtl2* DMR (IG-DMR) also exhibit paternal allele-specific bias in de novo methylation imprint establishment [41]. The maternally methylated *Snrpn*, *Zac1 and Peg1/Mest* DMRs are methylated faster in the maternal allele in growing oocytes [50], [72]. Similarly to the *H19/Igf2* ICR, allele-specific bias in chromatin composition of PGC origin may be responsible for providing epigenetic memory of the mother or father at these DMRs.

## Materials and Methods

The experiments involving mice had been approved by the IACUC of the City of Hope. Housing and care of the animals has been consistent with the Public Health Service Policy, the NIH “Guide for the Care and Use of Laboratory Animals” and the Animal Welfare Act.

### Purification of germ cells by flow cytometry

Male mice of the homozygous transgenic TgOG2 line [B6;CBA-Tg(Pou5f1-EGFP)2Mnn], which expresses the EGFP reporter gene specifically in germ cells [43] were mated to wild type females of FVB/NJ.CAST/Ei(N7) (CS), a distal chromosome 7 partial congenic strain [31] or to females carrying the *H19/Igf2* ICR CTCF site mutations (CTCFm) where the mutatant allele was derived from the 129SI/ImJ strain [31]. Pregnant females were sacrificed and from the fetuses female or male gonads were isolated and dispersed according to Buehr and McLaren [73]. Isolates were placed into 0.15 ml of trypsin-EDTA, incubated for 20 min at 37C° then dissociated into a single cell suspension. A total of 0.3 ml of 25% (v/v) fetal bovine serum in medium M2 [74] was added before flow cytometry. Cell suspensions were analyzed and sorted on a MoFlo flow cytometer (Beckman Coulter, Fort Collins, CO). Data were acquired using 488 nm excitation from an Innova-306 Argon laser (Coherrent, Santa Clara, CA) at 500 mW. EGFP emission was measured through a 530DF30 filter (Omega Optical, Brattleboro, VT).

### Bisulfite genomic sequencing

Fetal germ cells were flow-sorted, collected by centrifugation and embedded into agarose beads. Bisulfite sequencing of the ICR A region was done as before [31] according to Olek [51]. The average number of germ cells used per bisulfite reaction was 20,000. The range was between 1,200 and 27,000.

### Chromatin Immunoprecipitation

Chromatin preparation from 129 X CS primary MEFs was done as described earlier [34]. Chromatin was prepared from flow-sorted fetal germ cells similarly with modifications. We used chromatin from 100,000 cells per ChIP estimated by the number of sorted EGFP+ cells. We formaldehyde-crosslinked the chromatin in suspension for 2 min, stopped crosslinking by adding glycine, washed the cell pellet in PBS and resuspended the cells in M2 for flow cytometry. After sorting we resuspended the germ cells in lysis buffer, snap froze the chromatin aliquots and kept them deep frozen until sufficient quantities were obtained for several immunoprecipitations. We thawed the chromatin aliquots, sheared the chromatin by sonication and performed ChIP with different antibodies. The following antibodies were used in the chromatin immunoprecipitation (ChIP) assays: anti CTCF, 07-729; anti-dimethyl-histone H3 (Lys4), 07-030; anti-trimethyl-histone H3 (Lys9), 17–625; were purchased from Millipore and nonspecific IgG, sc-2027; was from Santa Cruz Biotechnology. The chromatin immunoprecipitation was performed as described previously [34] with minor modifications. Pre-blocked A/G beads from Santa Cruz (Cat#sc-2003) were used.

### Real-time PCR

Real-time PCR was performed to measure the region-specific overall ChIP enrichment levels at the *H19*-*Igf2* ICR as described [34].

### Analysis of allele-specific histone enrichment

To measure allele-specific chromatin differences we used the MALDI-TOF allelotyping analysis method from Sequenom [52] as we have done earlier [36]. Mass spectrometry was performed to quantify the extended SNuPE primers based on the differences in molecular mass between alleles. SNPs for the *H19*-*Igf2* region were obtained by DNA sequencing of inbred 129S1 (129) and CAST/Ei (CS) at specific regions of interest as described [34] or were provided by the introduced mutations [31]. Polymerase chain reaction and extension primers for the normal ICR (forward, reverse and UEP, respectively) were: SNuPE-*H19*-4kb: 5′-ACGTTGGATGTTGCGCCAAACCTAAAGAGC-3′; 5′-ACGTTGGATGAGGTACTGAACTTGGGTGAC-3′; 5′-CATTTGTGAATTCCAATACC-3′; SNuPE-*H19*-3kb: 5′-ACGTTGGATGACACTTGTGTTTCTGGAGGG-3′; 5′-ACGTTGGATGATGCCTTCCTATAGTGAGCC-3′; 5′-AAGGGGTCCCTTTGGTC-3′. Polymerase chain reaction and extension primers for the CTCF site mutant ICR (forward, reverse and UEP, respectively) were: SNuPE-CTCFm1#2: 5′-ACGTTGGATGCTTTAGGTTTGGCGCAATCG-3′; 5′-ACGTTGGATGCGTCTGCTGAATCAGTTGTG-3′; 5′-CGCAATCGATTTTGCTG-3′; SNuPE-CTCFm3#1: 5′-ACGTTGGATGGCTGTTATGTGCAACAAGGG-3′; 5′-ACGTTGGATGTGGGCCACGATATATAGGAG-3′; 5′-AAGGGAACGGATGCTAC-3′.

## Supporting Information

Figure S1 Verification of the purity of fetal germ cell population. Fetal ovaries and testes were dissected from the OG2 transgenic mouse line [43] and dissociated by trypsin digestion. Germ cells were separated from gonadal somatic cells using flow-cytometry. Germ cells can be distinguished by their GFP expression from the Pou5f1 promoter. Male (m) and female (f) cells were stained with a germ cell-specific DDX4 antibody (Abcam ab13840-100), before and after flow cytometry at (A) 15.5 dpc and (B) 13.5 dpc. The number of DDX4+/EGFP+ cells was 153/153 (100%), 265/272 (97%), 124/127 (98%) and 209/217 (96%) in male 15.5 dpc, male 13.5 dpc, female 15.5 dpc and female 13.5 dpc germ cells, respectively, in the flow-sorted cell populations.
(1.13 MB PDF)

Figure S2 The KvDMR1 is unmethylated in the purified fetal germ cells. The maternally methylated KvDMR1 is known to be unmethylated in fetal germ cells and becomes methylated only after birth, in the growing oocytes. We find that, correctly, none of the chromosomes were methylated in fetal germ cells at 13.5, 15.5 and 17.5 dpc. Sex of the gonad is indicated to the right.
(0.27 MB PDF)

Figure S3 DNA methylation is absent at the ICR in the female germ line. Bisulfite sequencing results of primary oocytes from (A) CS X OG2 and (B) CTCFm X OG2 fetuses was analyzed. Other details are as in Figure 2.
(0.58 MB PDF)

Figure S4 Methylation dynamics of the CTCF site mutant paternally inherited ICR. Bisulfite sequencing was performed using prospermatogonia from OG2 X CTCFm fetuses. The paternally inherited allele is shown. Other details are as in Figure 2.
(0.26 MB PDF)

Figure S5 ChIP-SNuPE assays for the H19/Igf2 ICR. Representative mixing experiments are shown. Fourteen control DNA samples were processed in replicates along with the ChIP samples. (A) Sonicated 129 (representing OG2 SNPs) and CS genomic DNA were mixed in different % ratios (100∶0, 95∶5, 90∶10, 80∶20, 70∶30, 60∶40 and 50∶50, 40∶60, 30∶70, 20∶80, 10∶90, 5∶95, 0∶100) for the standard curves. 129 X CS true heterozygote DNA was used for skew correction. The components of the assays, quantifying DNA alleles in the H19/Igf2 ICR at −3 and −4 kb positions upstream of the H19 transcription start site are indicated in Figure 1. (B) Sonicated CTCFm and OG2 DNA were mixed similarly. CTCFm X OG2 true heterozygote DNA was used for skew correction. The components of the assays, quantifying DNA alleles in the H19/Igf2 ICR at mutant CTCF site 1 and 3 (again at about −3 kb and −4 kb positions upstream of the H19 transcription start site) are indicated to the right. Average measured ratios were plotted against the input ratios with standard deviations. The four assays were rigorously quantitative using small amounts (25 ng) of total DNA.
(0.30 MB PDF)

Figure S6 Validation of the ChIP-SNuPE assays for small numbers of cells. ChIP-SNuPE Sequenom assays are shown. (A) ChIP was performed using 100,000 MEF cells from the 129 X CS mouse cross. The ChIP-SNuPE assays specific for the ICR −4 kb and −3 kb regions (A and B) were used to quantitate the percent of the maternal (black) or paternal (grey) allele in the total immunoprecipitation or in the total input chromatin. The antibodies are indicated at the bottom (B) ChIP-SNuPE assays were performed on independent immunoprecipitated chromatin samples obtained with the nonspecific IgG antibody. Female and male germ cells from 14.5 dpc CS X OG2 fetuses were assessed. (C) ChIP-SNuPE assays using ChIP input samples from female or male CS X OG2 and CTCFm X OG2 germ cells at 14.5 dpc. Other details are as in Figure 4.
(0.32 MB PDF)

Figure S7 CTCF enrichment at the ICR in germ cell chromatin. Real-time PCR quantification of CTCF-bound H19/Igf2 ICR is shown at regions A (−4 kb) and B (−3 kb). Average precipitated copy numbers are plotted with standard deviations. The copy numbers were calculated based on known copy numbers of serial dilution of sheared genomic DNA run in parallel. 3 µl out of the total 25 µl ChIP DNA was used for real-time PCR. The numbers correspond to precipitation from 12,000 out of a total 100,000 cells. The CS X OG2 and CTCFm X OG2 germ cell ChIP values are much lower for the CTCF antibody (Millipore07-729) at 14.5 dpc than those obtained in the same number of 129 X CS MEF cells.
(0.24 MB PDF)

Figure S8 CTCF is not absent from germ cells at 12.5 and 14.5 dpc. Anti-CTCF antibody (Millipore07-729) staining (red) is at similar levels between gonadal germ cells (GFP positive) and somatic cells (GFP negative) at 12.5 and 14.5 dpc. DAPI signal indicates nuclei.
(0.05 MB PDF)

Figure S9 H3K4me2 ChIP intensities in germ cell chromatin real-time PCR results are shown for two sets of experiments at two ICR regions (A and B) as indicated above each graph. The CS X OG2 and CTCFm X OG2 germ cell ChIP precipitation values with the H3K4me2-specific antibody at 13.5 dpc and 14.5 dpc are comparable to those obtained of the same number of 129 X CS MEFs. The non-specific IgG had very low background in germ cells, just like in MEFs [36]. Other details are as in Figure S7.
(0.31 MB PDF)
