# Supplementary figures and images for: CTCF-Dependent Chromatin Bias Constitutes Transient Epigenetic Memory of the Mother at the H19-Igf2 Imprinting Control Region in Prospermatogonia
Source: PLoS Genet. 2010 Nov 24;6(11):e1001224. doi: 10.1371/journal.pgen.1001224 (PMC2991272; doi:10.1371/journal.pgen.1001224)

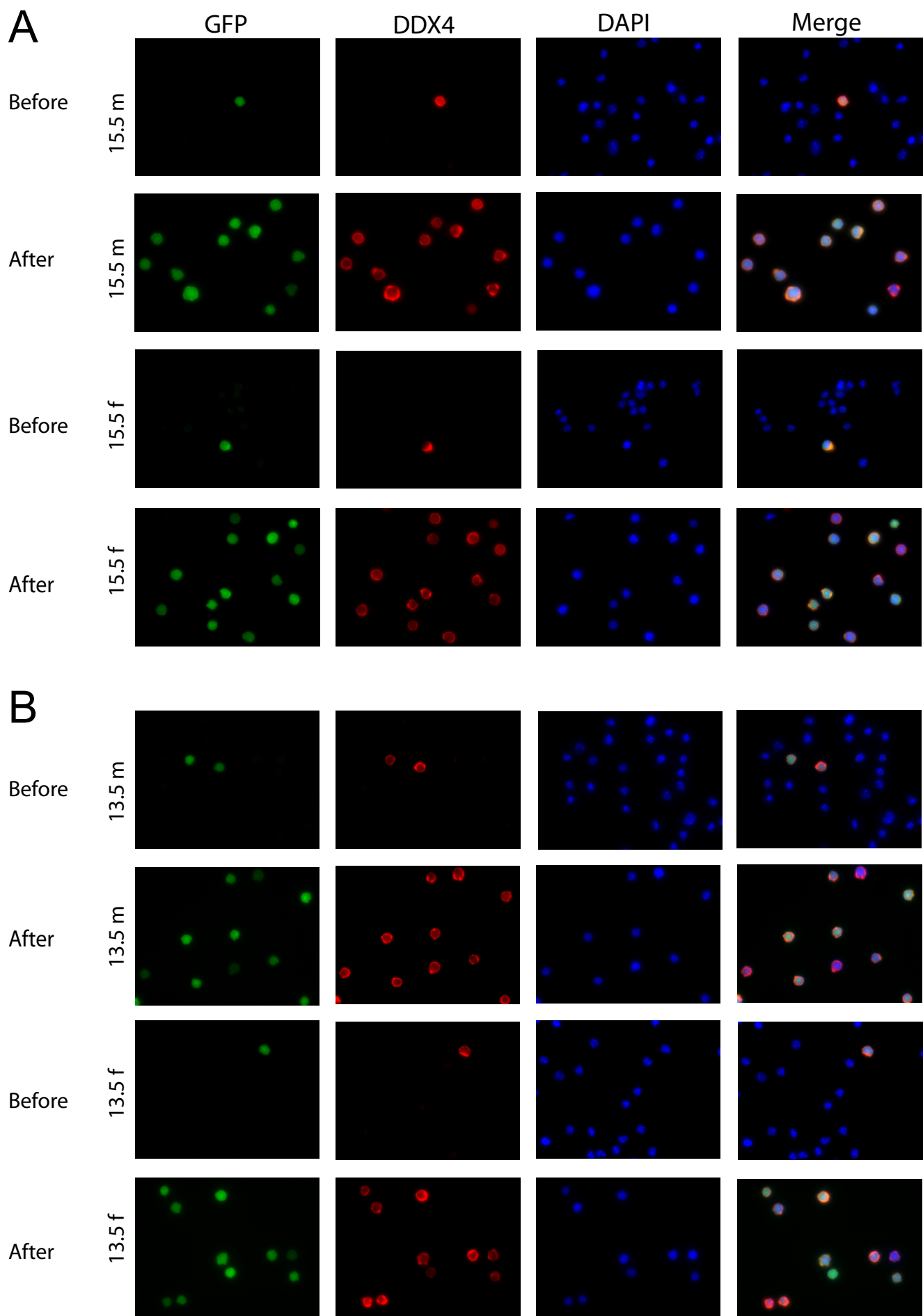

Supplement: Figure S1 — Verification of the purity of fetal germ cell population. Fetal ovaries and testes were dissected from the OG2 transgenic mouse line [43] and dissociated by trypsin digestion. Germ cells were separated from gonadal somatic cells using flow-cytometry. Germ cells can be distinguished by their GFP expression from the Pou5f1 promoter. Male (m) and female (f) cells were stained with a germ cell-specific DDX4 antibody (Abcam ab13840-100), before and after flow cytometry at (A) 15.5 dpc and (B) 13.5 dpc. The number of DDX4+/EGFP+ cells was 153/153 (100%), 265/272 (97%), 124/127 (98%) and 209/217 (96%) in male 15.5 dpc, male 13.5 dpc, female 15.5 dpc and female 13.5 dpc germ cells, respectively, in the flow-sorted cell populations. (1.13 MB PDF) [file pgen.1001224.s001.pdf]

KvDMR1

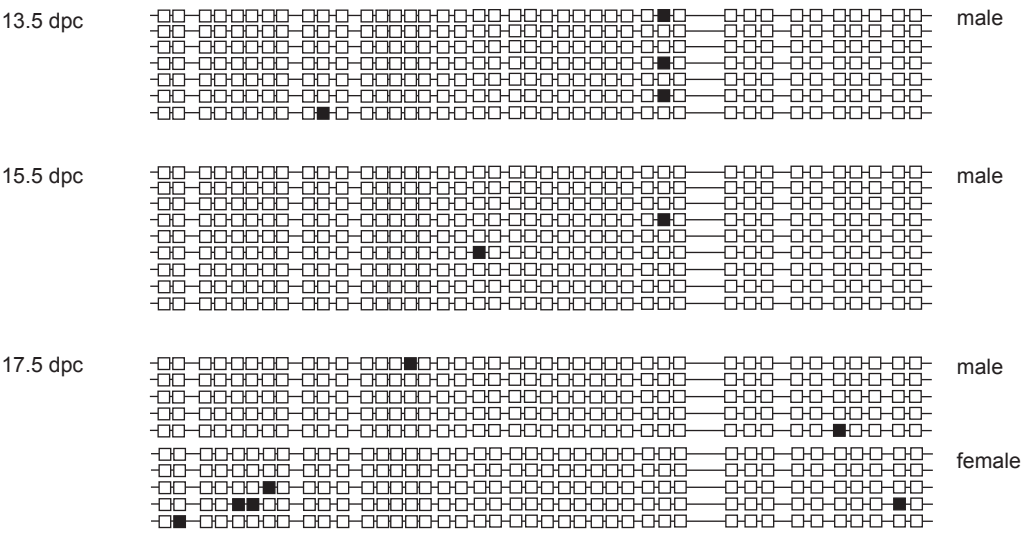

Figure S2 Lee et al.

Supplement: Figure S2 — The KvDMR1 is unmethylated in the purified fetal germ cells. The maternally methylated KvDMR1 is known to be unmethylated in fetal germ cells and becomes methylated only after birth, in the growing oocytes. We find that, correctly, none of the chromosomes were methylated in fetal germ cells at 13.5, 15.5 and 17.5 dpc. Sex of the gonad is indicated to the right. (0.27 MB PDF) [file pgen.1001224.s002.pdf]

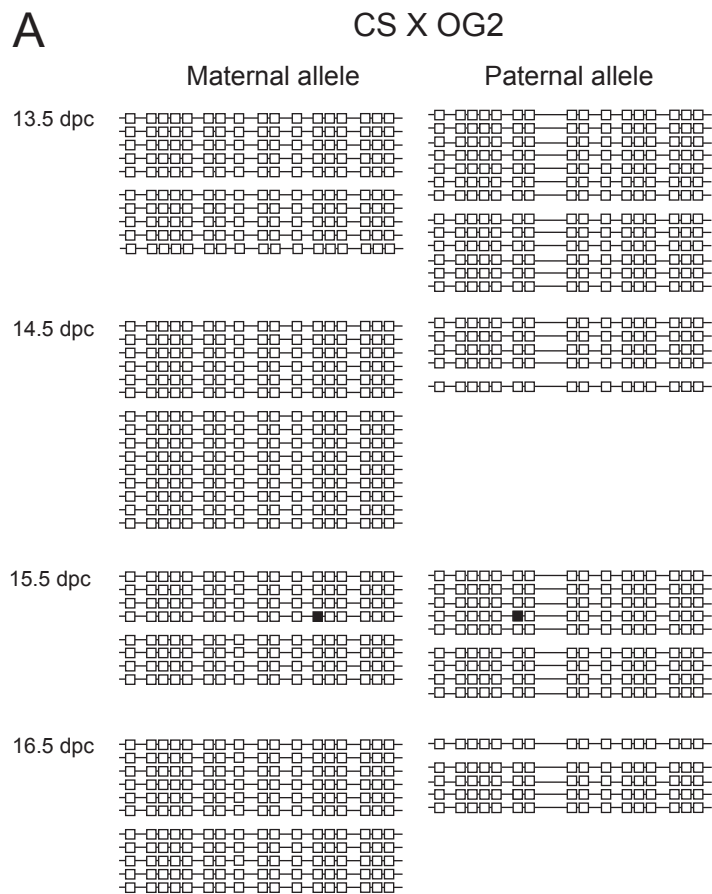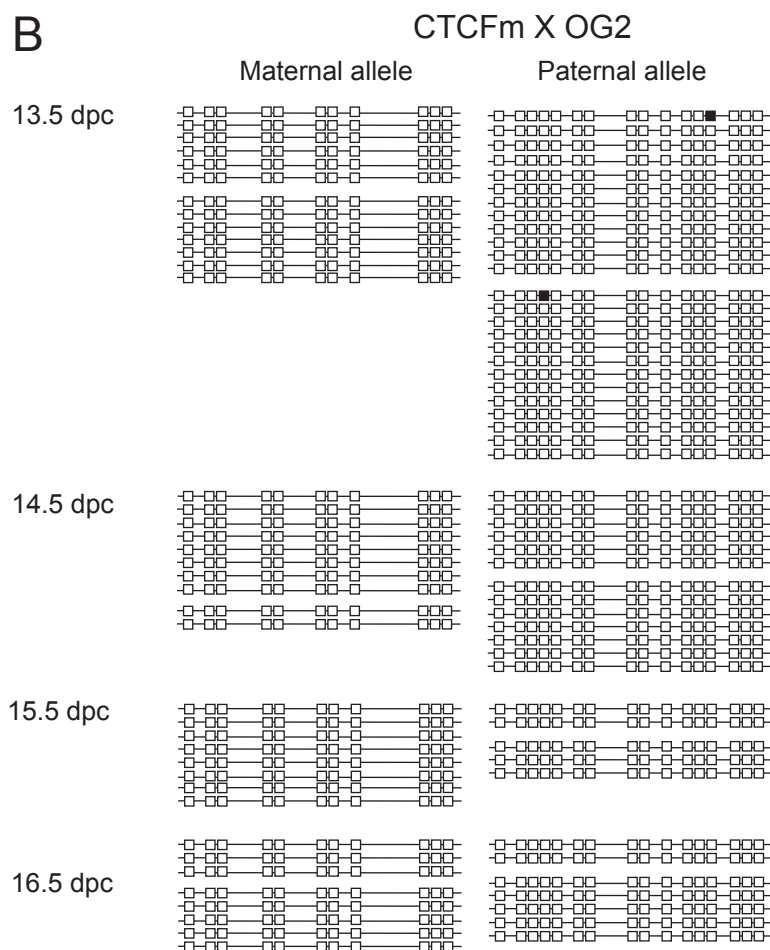

Figure S3 Lee et al.

Supplement: Figure S3 — DNA methylation is absent at the ICR in the female germ line. Bisulfite sequencing results of primary oocytes from (A) CS X OG2 and (B) CTCFm X OG2 fetuses was analyzed. Other details are as in Figure 2. (0.58 MB PDF) [file pgen.1001224.s003.pdf]

15.5 dpc

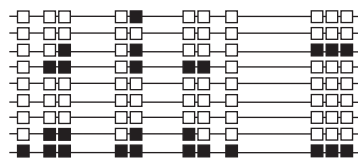

26% (26/99)

16.5 dpc

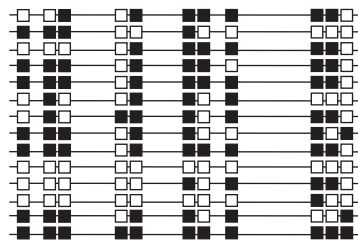

55% (84/154)

Figure S4 Lee et al.

Supplement: Figure S4 — Methylation dynamics of the CTCF site mutant paternally inherited ICR. Bisulfite sequencing was performed using prospermatogonia from OG2 X CTCFm fetuses. The paternally inherited allele is shown. Other details are as in Figure 2. (0.26 MB PDF) [file pgen.1001224.s004.pdf]

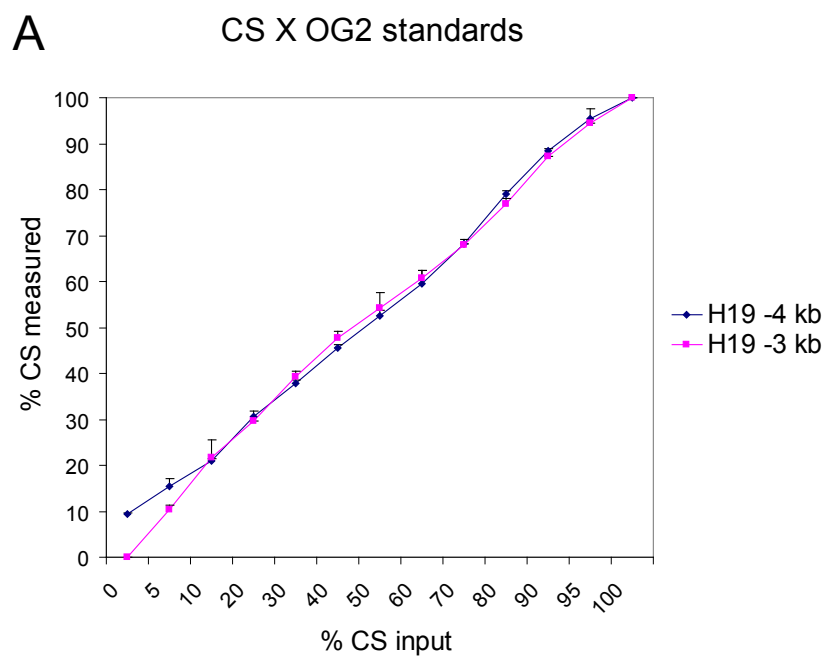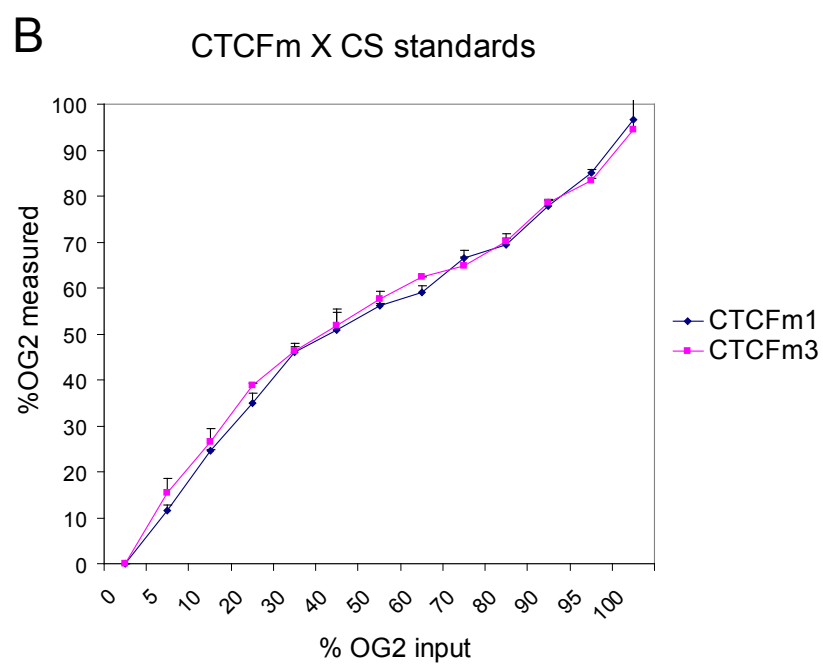

Figure S5 Lee et al.

Supplement: Figure S5 — ChIP-SNuPE assays for the H19/Igf2 ICR. Representative mixing experiments are shown. Fourteen control DNA samples were processed in replicates along with the ChIP samples. (A) Sonicated 129 (representing OG2 SNPs) and CS genomic DNA were mixed in different % ratios (100∶0, 95∶5, 90∶10, 80∶20, 70∶30, 60∶40 and 50∶50, 40∶60, 30∶70, 20∶80, 10∶90, 5∶95, 0∶100) for the standard curves. 129 X CS true heterozygote DNA was used for skew correction. The components of the assays, quantifying DNA alleles in the H19/Igf2 ICR at −3 and −4 kb positions upstream of the H19 transcription start site are indicated in Figure 1. (B) Sonicated CTCFm and OG2 DNA were mixed similarly. CTCFm X OG2 true heterozygote DNA was used for skew correction. The components of the assays, quantifying DNA alleles in the H19/Igf2 ICR at mutant CTCF site 1 and 3 (again at about −3 kb and −4 kb positions upstream of the H19 transcription start site) are indicated to the right. Average measured ratios were plotted against the input ratios with standard deviations. The four assays were rigorously quantitative using small amounts (25 ng) of total DNA. (0.30 MB PDF) [file pgen.1001224.s005.pdf]

A

MEF 129 X CS

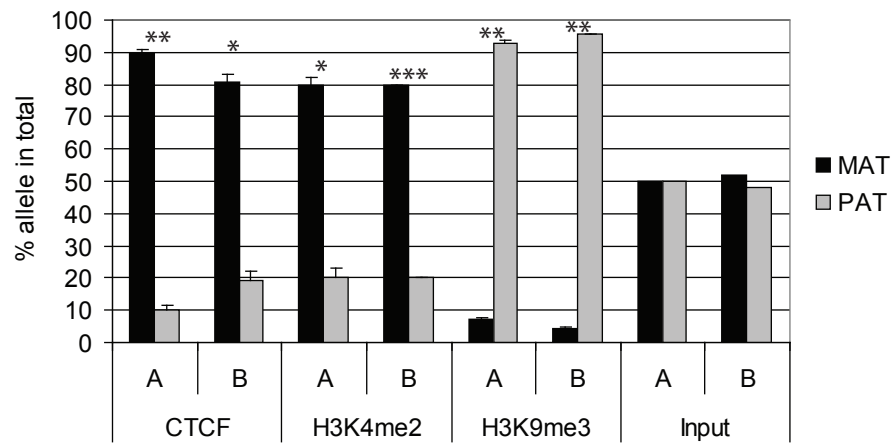

B

Nonspecific IgG

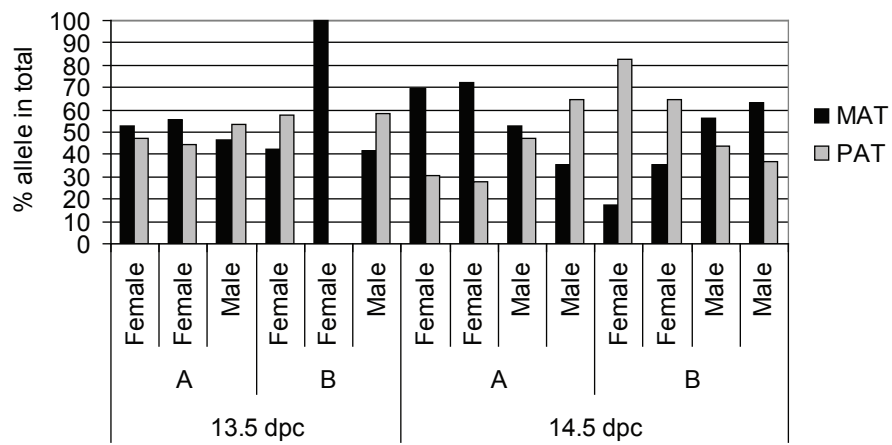

C

Input 14.5 dpc

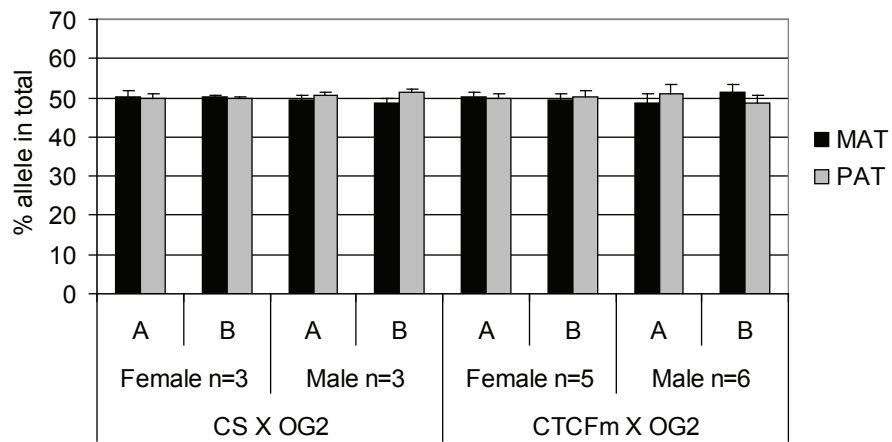

Figure S6 Lee et al.

Supplement: Figure S6 — Validation of the ChIP-SNuPE assays for small numbers of cells. ChIP-SNuPE Sequenom assays are shown. (A) ChIP was performed using 100,000 MEF cells from the 129 X CS mouse cross. The ChIP-SNuPE assays specific for the ICR −4 kb and −3 kb regions (A and B) were used to quantitate the percent of the maternal (black) or paternal (grey) allele in the total immunoprecipitation or in the total input chromatin. The antibodies are indicated at the bottom (B) ChIP-SNuPE assays were performed on independent immunoprecipitated chromatin samples obtained with the nonspecific IgG antibody. Female and male germ cells from 14.5 dpc CS X OG2 fetuses were assessed. (C) ChIP-SNuPE assays using ChIP input samples from female or male CS X OG2 and CTCFm X OG2 germ cells at 14.5 dpc. Other details are as in Figure 4. (0.32 MB PDF) [file pgen.1001224.s006.pdf]

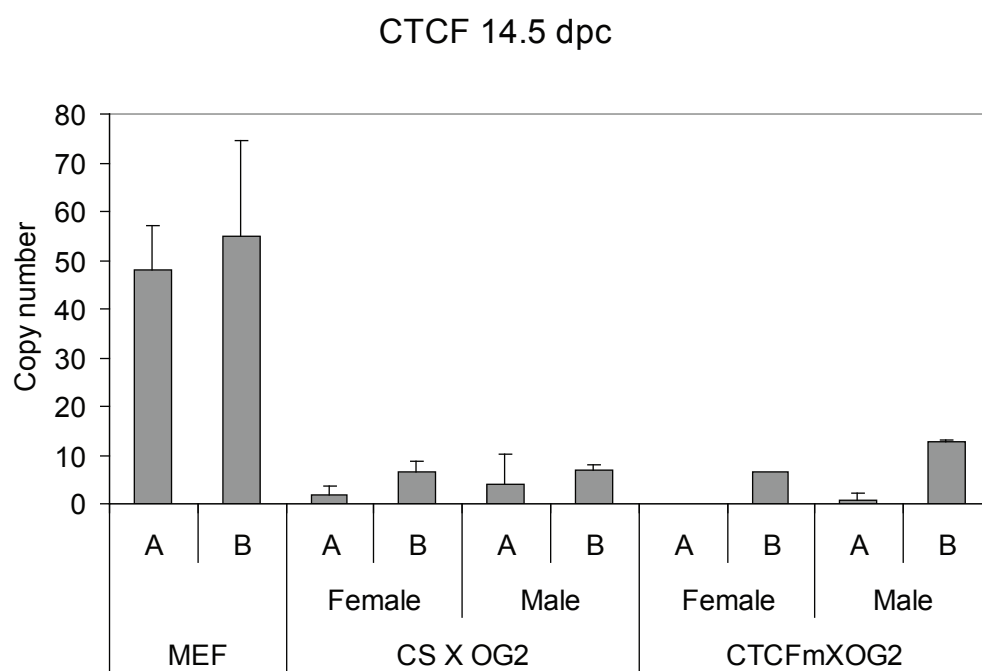

Figure S7 Lee et al.

Supplement: Figure S7 — CTCF enrichment at the ICR in germ cell chromatin. Real-time PCR quantification of CTCF-bound H19/Igf2 ICR is shown at regions A (−4 kb) and B (−3 kb). Average precipitated copy numbers are plotted with standard deviations. The copy numbers were calculated based on known copy numbers of serial dilution of sheared genomic DNA run in parallel. 3 µl out of the total 25 µl ChIP DNA was used for real-time PCR. The numbers correspond to precipitation from 12,000 out of a total 100,000 cells. The CS X OG2 and CTCFm X OG2 germ cell ChIP values are much lower for the CTCF antibody (Millipore07-729) at 14.5 dpc than those obtained in the same number of 129 X CS MEF cells. (0.24 MB PDF) [file pgen.1001224.s007.pdf]

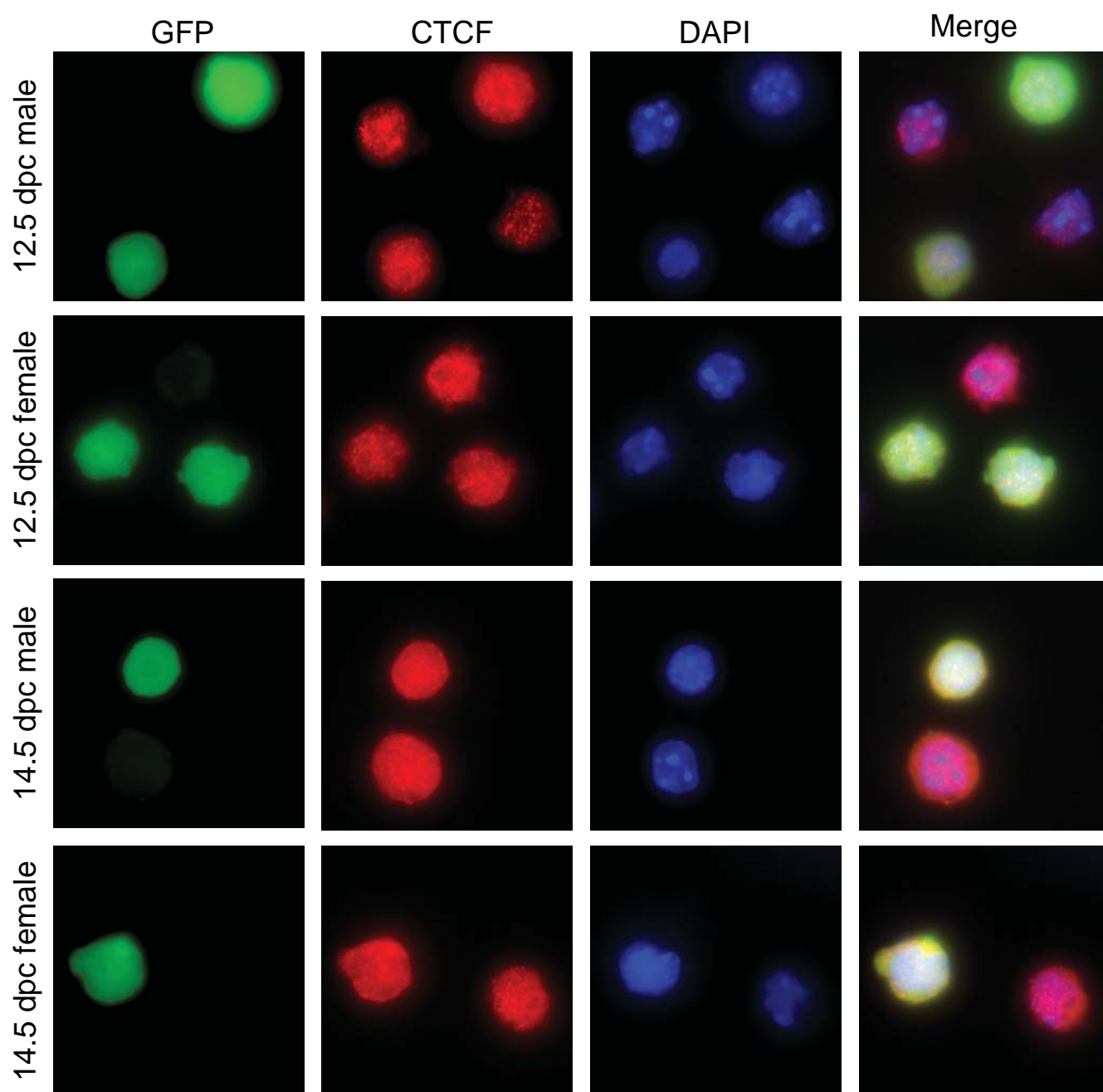

Figure S8 Lee et al.

Supplement: Figure S8 — CTCF is not absent from germ cells at 12.5 and 14.5 dpc. Anti-CTCF antibody (Millipore07-729) staining (red) is at similar levels between gonadal germ cells (GFP positive) and somatic cells (GFP negative) at 12.5 and 14.5 dpc. DAPI signal indicates nuclei. (0.05 MB PDF) [file pgen.1001224.s008.pdf]

### H3K4me2 Region A

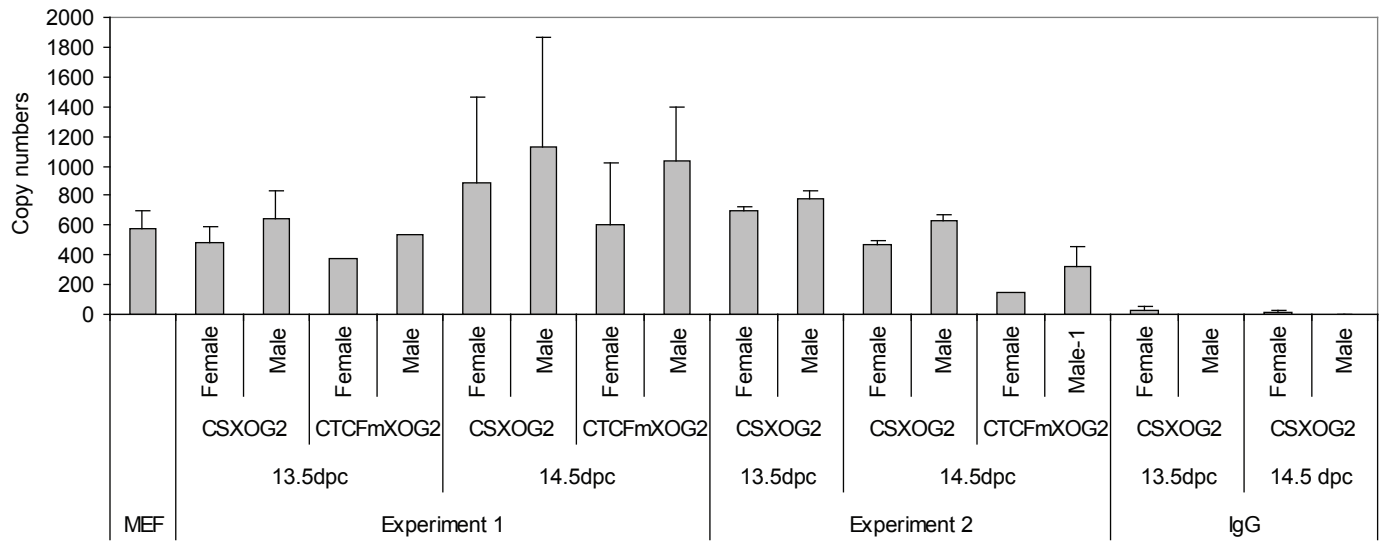

### H3K4me2 Region B

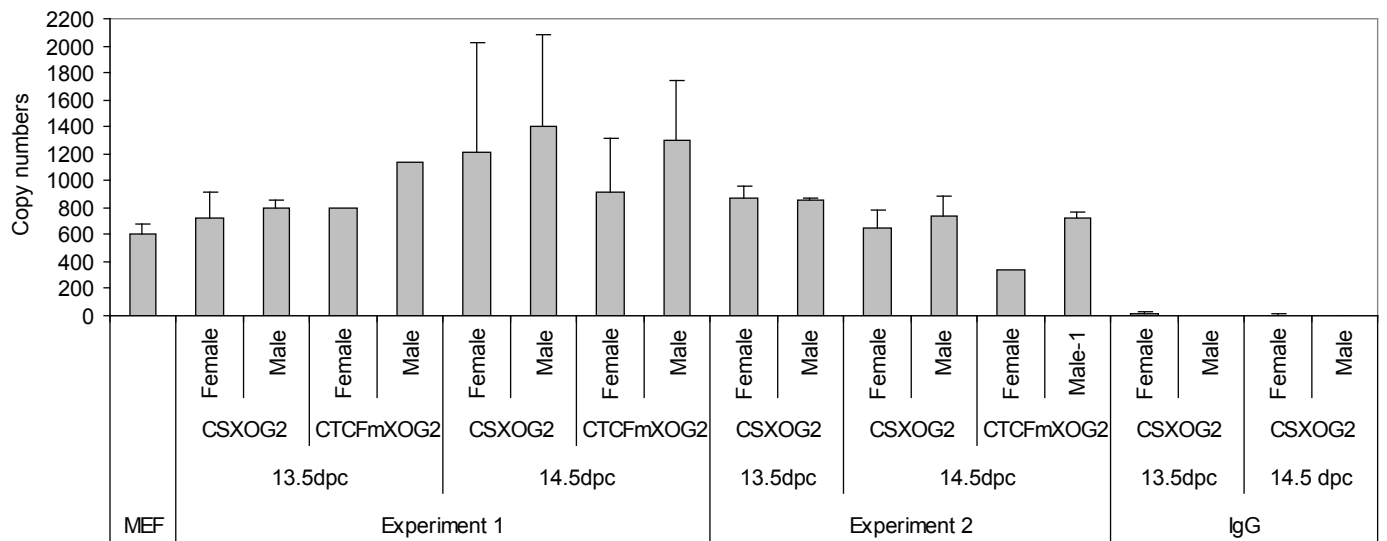

Figure S9 Lee et al.

Supplement: Figure S9 — H3K4me2 ChIP intensities in germ cell chromatin real-time PCR results are shown for two sets of experiments at two ICR regions (A and B) as indicated above each graph. The CS X OG2 and CTCFm X OG2 germ cell ChIP precipitation values with the H3K4me2-specific antibody at 13.5 dpc and 14.5 dpc are comparable to those obtained of the same number of 129 X CS MEFs. The non-specific IgG had very low background in germ cells, just like in MEFs [36]. Other details are as in Figure S7. (0.31 MB PDF) [file pgen.1001224.s009.pdf]
